# Micro-hepatocellular carcinoma with bile duct tumor thrombus mimicking intrahepatic intraductal papillary neoplasm of the bile duct: a case report

**DOI:** 10.1186/s40792-023-01646-3

**Published:** 2023-04-30

**Authors:** Takatsugu Matsumoto, Takayuki Shimizu, Shun Sato, Genki Tanaka, Takamune Yamaguchi, Kyung-Hwa Park, Yuhki Sakuraoka, Takayuki Shiraki, Shozo Mori, Yukihiro Iso, Takehiko Nemoto, Keiichi Kubota, Yumi Nozawa, Kazuyuki Ishida, Taku Aoki

**Affiliations:** 1 Department of Hepato-Biliary-Pancreatic Surgery, Dokkyo Medical University, 880 Kitakobayashi, Mibu, Tochigi 321-0293 Japan; 2 Department of Surgery, Tohto Bunkyo Hospital, Bunkyo-ku, Tokyo, Japan; 3 Department of Diagnostic Pathology, Dokkyo Medical University, Mibu, Tochigi, Japan

**Keywords:** Bile duct tumor thrombus, Hepatocellular carcinoma, Liver resection

## Abstract

**Background:**

Microhepatocellular carcinoma with a gross bile duct tumor thrombus is extremely rare, making the correct preoperative diagnosis difficult.

**Case presentation:**

A 78-year-old man was referred to our department for close examination of a liver tumor that was incidentally detected using ultrasonography. Blood tests revealed normal levels of tumor markers. Abdominal ultrasonography showed a 2-cm-sized hyperechoic mass with indistinct borders and hypoechoic margins at the origin of the right hepatic duct. Dynamic computed tomography showed a tumor with arterial phase predominance, a heterogeneous contrast effect, and prolonged enhancement. Cystic structures were observed in the tumors. In addition, localized dilatation of the caudate lobe bile duct was observed near the tumor. Cholangiography showed that the common bile duct, right and left hepatic ducts, and secondary branches did not have dilatation or stenosis. Biopsies of the bile duct revealed no malignancy. Under suspicion of intrahepatic intraductal papillary neoplasm of the bile duct, right hemi-hepatectomy was performed. The extrahepatic bile duct was preserved, because no tumor was found at the margin of the right hepatic duct during intraoperative frozen diagnosis. Macroscopically, the lesion was an 18 × 15 mm tumor occupying a dilated intrahepatic bile duct near the right hepatic duct, with a soft, fine papillary tumor. Based on morphology and immunostaining, tumor matched with moderately differentiated hepatocellular carcinoma. In addition, a 2 mm-sized hepatocellular carcinoma was observed in the liver parenchyma near the bile duct, where the tumor was located.

**Conclusions:**

Based on these findings, the patient was diagnosed with small hepatocellular carcinoma with a gross bile duct tumor thrombus. The cystic part seen on the preoperative images was considered as a gap between the bile duct and the tumor thrombus. The patient recovered well with no signs of recurrence 20 months after surgery.

## Background

Liver cancer is the sixth most common malignancy and second leading cause of cancer-related deaths in men worldwide [1]. Hepatocellular carcinoma (HCC) is the most common type of primary liver cancer, comprising 75–85% of cases [1]; however, HCC with bile duct tumor thrombus (BDTT) is uncommon, with an incidence between 0.53% and 12.9% [2–5]. However, most of these cases showed radiologically identifiable primary tumors. Herein, we present an unusual case of HCC of microscopic origin with macroscopic BDTT that mimicked intrahepatic intraductal papillary neoplasm of the bile duct in which hepatic resection was performed.

## Case presentation

A 78-year-old man was referred to our department for close examination of a liver tumor. Abdominal ultrasonography revealed a 2-cm-sized hyperechoic mass with indistinct borders at the origin of the right hepatic duct. The patient had a medical history of diabetes mellitus, vasospastic angina pectoris, and transurethral resection for bladder carcinoma. Physical examination results were unremarkable. Laboratory data showed slightly elevated hemoglobin A1c levels. Anti-Hepatitis B surface- and core antibodies were positive. However, the result of serum hepatitis B virus-polymerase chain reaction test was negative. All tumor markers, including carcinoembryonic antigen (CEA), carbohydrate antigen 19-9 (CA19-9), alpha-fetoprotein (AFP) and protein induced by vitamin K absence or antagonist-II (PIVKAII), were within the normal ranges. The retention rate of indocyanine green at 15 min was 10%. The Child–Pugh score was 5 points (Table 1).

**Table 1:** Laboratory data on admission

AST	(8–38, U/l)	20	WBC	(3200–8500/mm^3^)	5200
ALT	(4–44, U/l)	18	Hb	(11.0–14.8, g/dl)	16.6
ALP	(104–338, U/l)	61	Plt	(16.4–35.8, 10^4^/μl)	20.4
LDH	(106–211, U/l)	200	PT	(70, %)	107
T-Bil	(0.1–1.0, mg/dl)	0.8	APTT	(sec)	34.2
D-Bil		0.3			
TP	(6.5–8.1, g/dl)	7.3	Fbg	(150–400, mg/dl)	300
Alb	(3.9–4.9, g/dl)	4.6	CRP	(− 0.3, mg/dl)	0.1
Na	(135–151, mEq/l)	141			
K	(3.3–4.8, mEq/l)	3.9	ICGR15	(− 10, %)	10
Cl	(98–108, mEq/l)	105	CEA	(− 5.0, ng/ml)	0.83
UN	(7–21, mg/dl)	24.6	CA19-9	(− 37, U/ml)	6.60
Cre	(0.4–0.8, mg/dl)	1.01	AFP	(− 25, U/ml)	5.2
HbA1c	(− 5.7, %)	6.8	PIVKA-II	(− 30, U/ml)	2.4
			HBsAg		0.1
			HBsAb		1179.3
			HBcAb		5.12
			HCVAb		0.04

Underlines indicate anomalous value

*APTT* Activated partial thromboplastin time, *Alb* albumin, *ALP* alkaline phosphatase, *ALT* alanine aminotransferase, *AFP* alpha-fetoprotein, *UN* blood urea nitrogen, *CEA* carcinoembryonic antigen, *CA19-9* carbohydrate antigen 19-9, *CRP* C-reactive protein, *Cre* creatinine, *D-bil* direct bilirubin, *Fbg* fibrinogen, *HbA1c* glycated hemoglobin, *Hb* hemoglobin, *ICGR15* indocyanine green retention rate at 15 min, *LDH* lactate dehydrogenase, *Plt* platelet, *PT* prothrombin time, *PIVKA-II* protein induced by vitamin K absence or antagonist-II, *AST* aspartate aminotransferase, *T-bil* total bilirubin, *TP* total protein, and *WBC* white blood cell

**Fig. 1:**
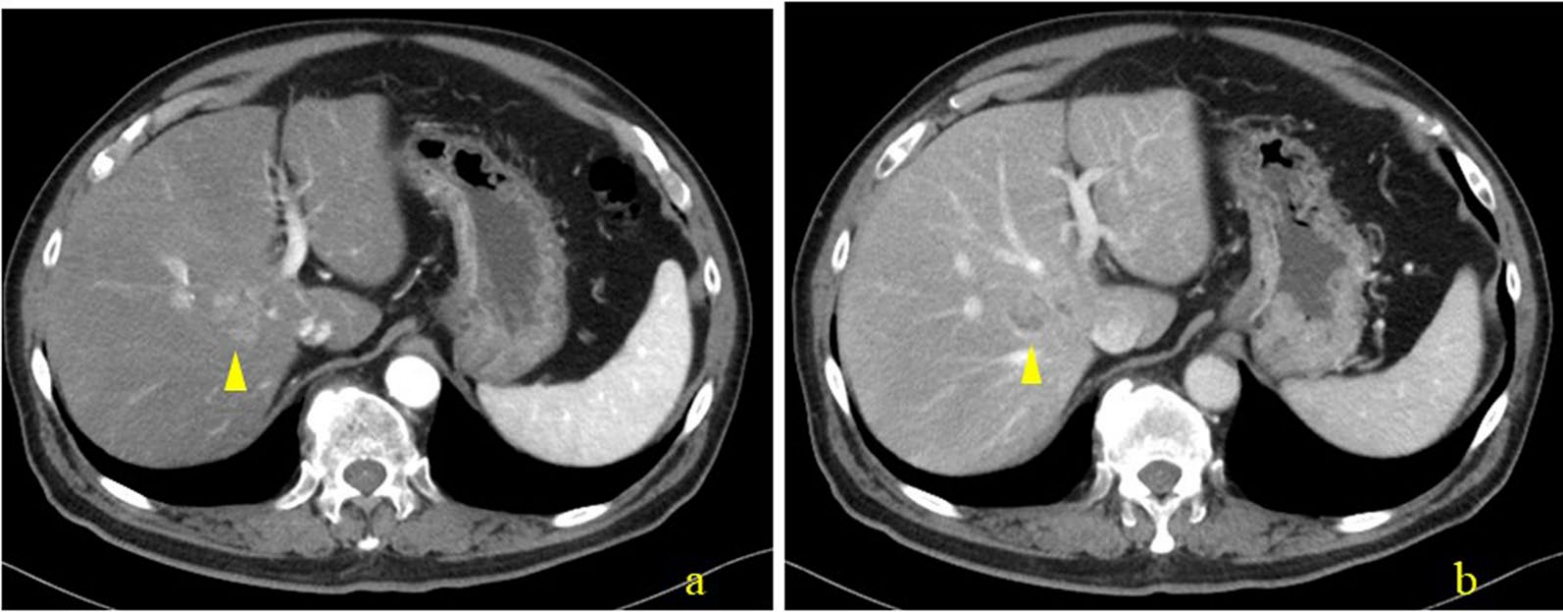
Abdominal contrast-enhanced computed tomography. **a** Tumor showed a heterogeneous contrast effect in the arterial phase, located at the hepatic hilum, slightly cephalad, and dorsal to the right hepatic duct (arrowhead). **b** Tumor showed prolonged enhancement at the late phase, accompanied partly by a low attenuated component mimicking cyst-like structures

**Fig. 2:**
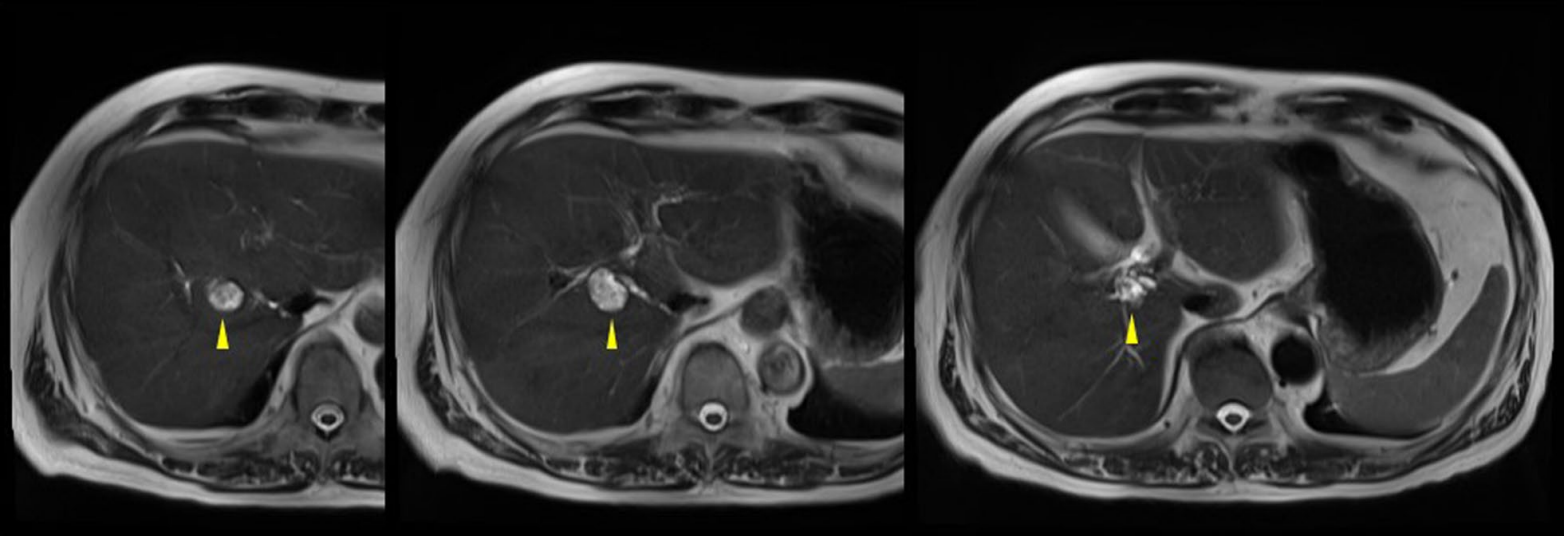
Magnetic resonance imaging. Magnetic resonance imaging showed high intensity on T2 weighted images with a heterogeneous low-intensity area in the lumen (arrowhead)

**Fig. 3:**
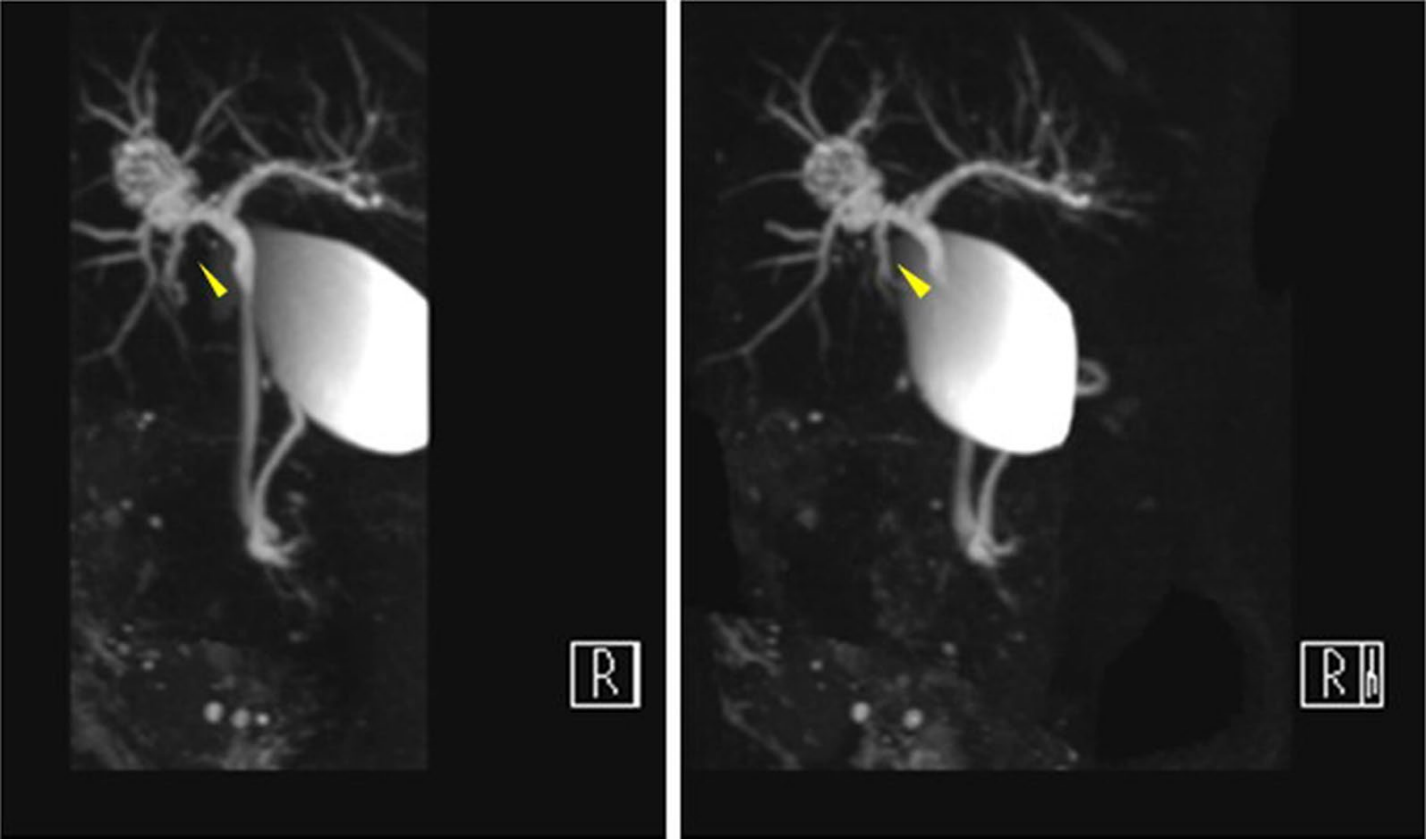
Magnetic resonance cholangiopancreatography. A cystic lesion located adjacent to the right hepatic hilum was observed. In addition, the bile duct of the right caudate lobe was significantly dilated (arrowhead), suggesting that the tumor originates from the origin of the bile duct of the right caudate lobe

Contrast-enhanced computed tomography (CECT) revealed a tumor, 2 cm in size, in the hilar region, slightly cephalad, and dorsal to the right hepatic duct (Fig. 1). The tumor showed a heterogeneous contrast effect in the arterial phase, with prolonged enhancement, accompanied partly by a low-attenuation component mimicking cystic structures. In addition, localized dilatation of the caudate lobe bile duct was observed near the tumor. Magnetic resonance imaging (MRI) showed high intensity on T2 weighted images dorsal to the origin of the right hepatic duct with a heterogeneous low-intensity area in the lumen (Fig. 2). The tumor showed mild diffusion restriction on diffusion-weighted imaging. The tumor showed a low signal intensity on the hepatocyte phase of the contrast-enhanced MRI. Magnetic resonance cholangiopancreatography demonstrated the existence of a cystic lesion located adjacent to the right hepatic hilum. In addition, the bile duct of the right caudate lobe was found to be significantly dilated, suggesting that the tumor originates from the origin of the bile duct of the right caudate lobe (Fig. 3). Moreover, the tumor appeared to fill the bile duct without evidence of invasion into the liver parenchyma. The tumor was also suspected to be a papillary or multicystic growth pattern. Endoscopic retrograde cholangiography revealed no abnormal findings and failed to detect any tumor. Biopsies of the hilar hepatic duct and the common bile duct showed no malignant tissue.

Based on these findings, right hemi-hepatectomy was performed, suspecting intrahepatic intraductal papillary neoplasm of the bile duct. As the estimated future remnant liver volume after right hemihepatectomy was 34% of the total liver volume, portal vein embolization of the right branch was performed prior to the liver resection to prevent postoperative liver failure.

The extrahepatic bile duct was preserved, because no tumor was found at the margin of the right hepatic duct during intraoperative frozen diagnosis. Lymphadenectomy was not performed in this case, because it is not routinely performed for IPNB.

**Fig. 4:**
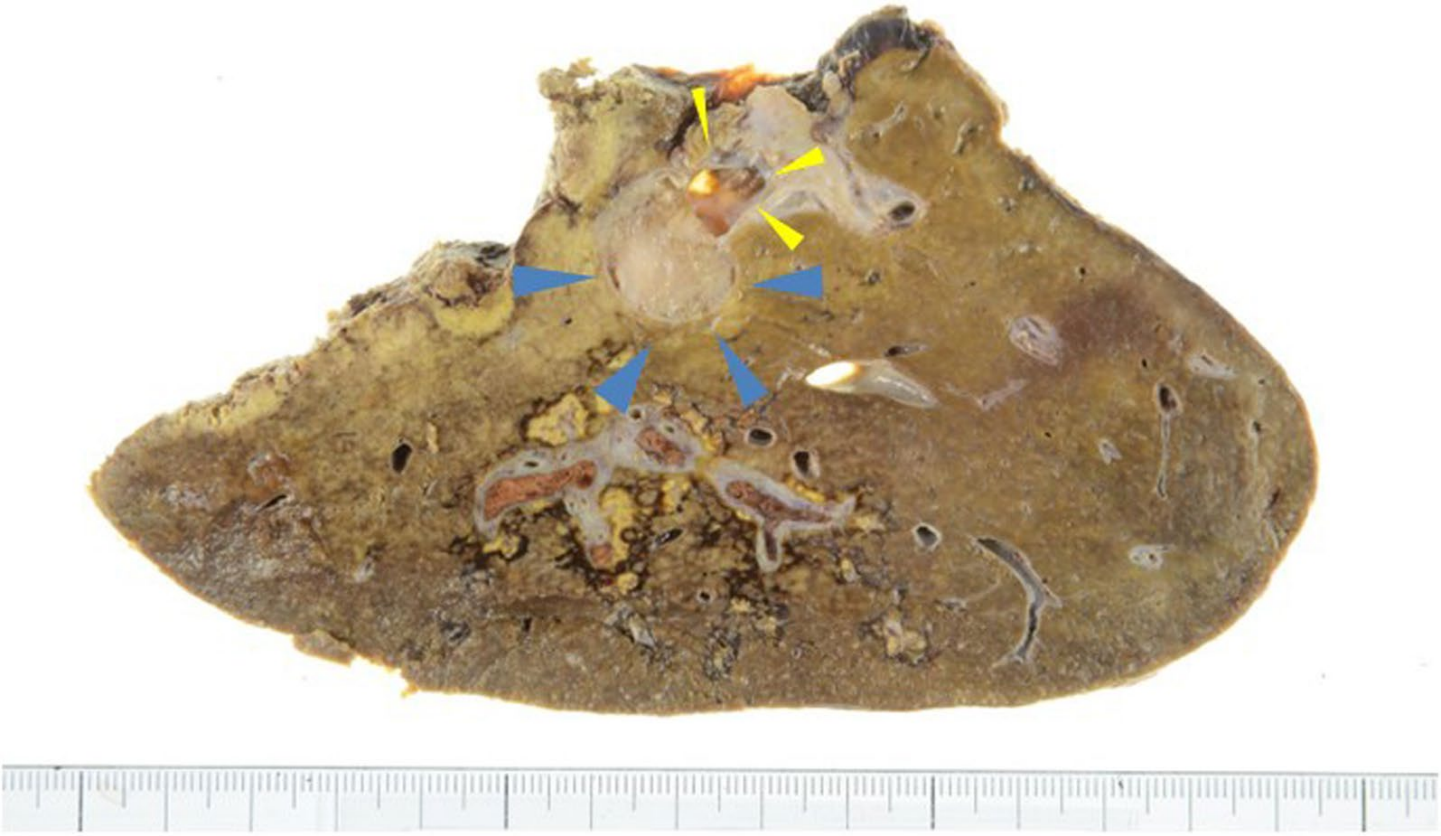
Macroscopic findings. The lesion was an 18 × 15 mm tumor occupying a dilated intrahepatic bile duct near the right hepatic duct (yellow arrowhead), with a soft and fine papillary tumor (blue arrowhead)

**Fig. 5:**
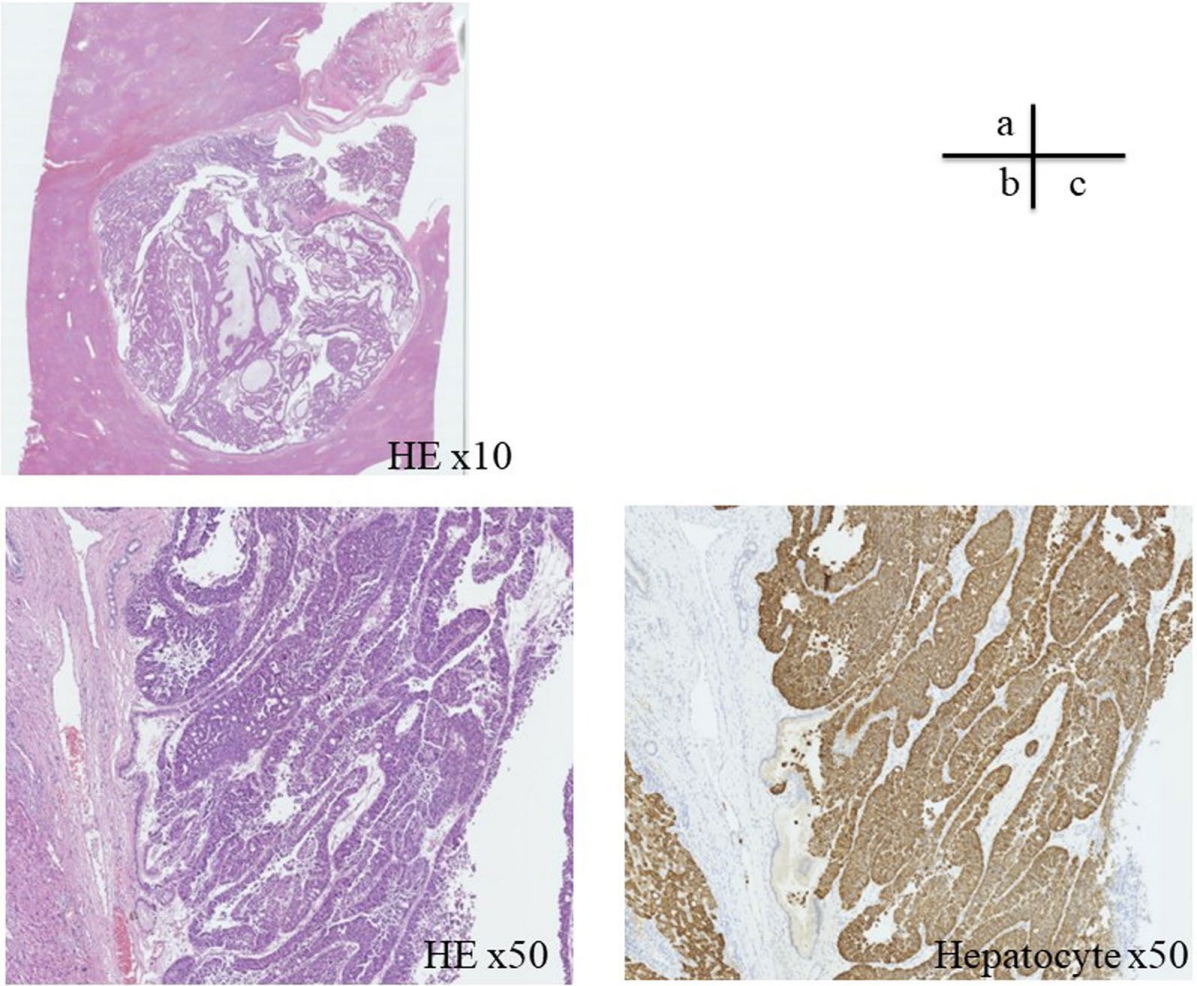
Microscopic findings. **a** Tumor filled the bile duct, proliferating in a nearly cord-like ductal structure. **b** Tumor grew in a nearly cord-like tubular structure and showed carcinoma-equivalent atypia with finely granular, highly acidophilic, abundant cytoplasm, and irregular round nuclei with agglutinated chromatin, which were consistent with moderately differentiated HCC. **c** Immunostaining also showed that the tumor cells were hepatocyte-positive, indicating hepatocellular carcinoma

**Fig. 6:**
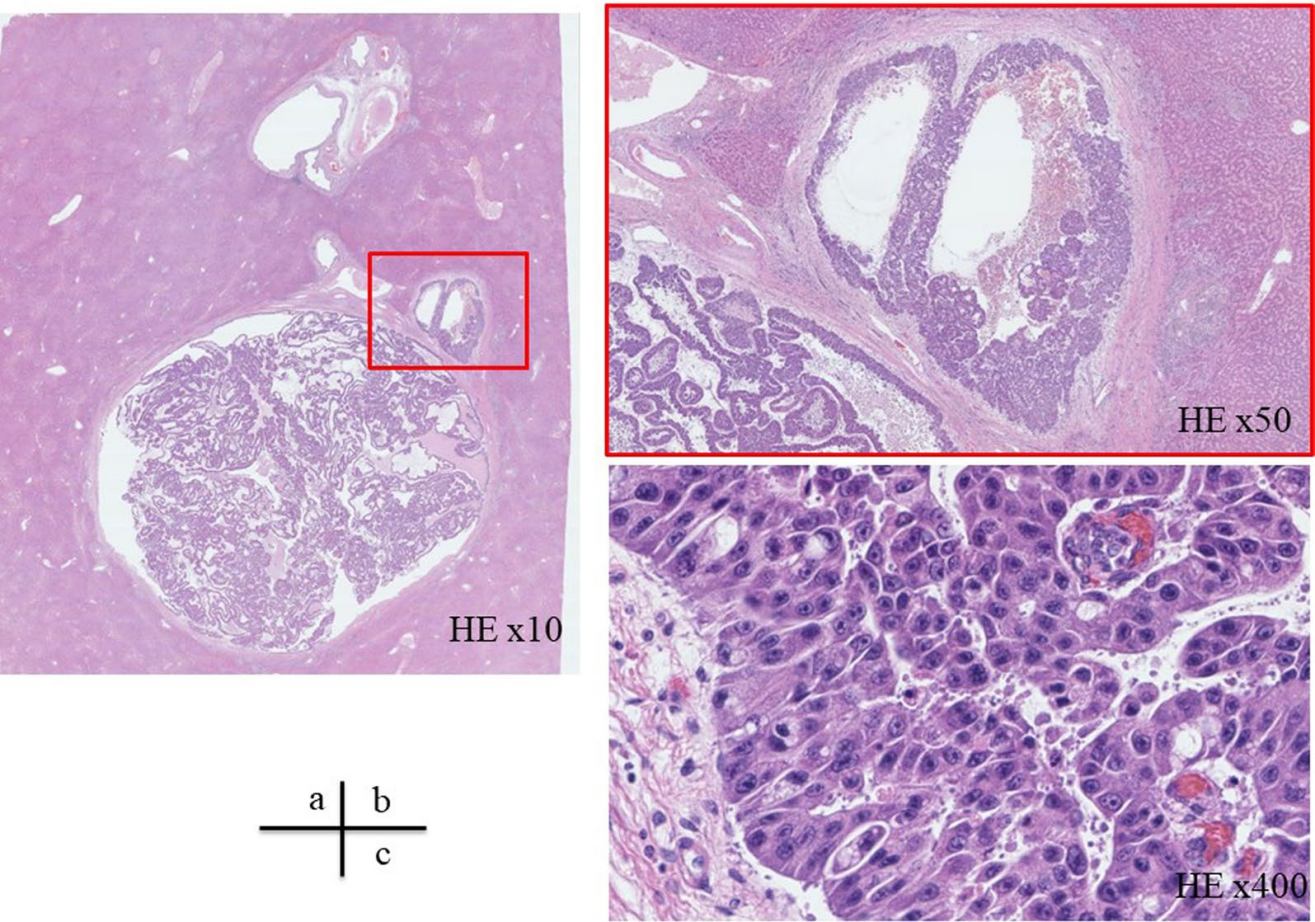
M**i**croscopic findings [2]. Hepatocellular carcinoma, 2 mm in size with stromal invasion, was present in the vicinity of the bile duct. **a** HE × 10. **b** HE × 50. **c** HE × 400

**Fig. 7:**
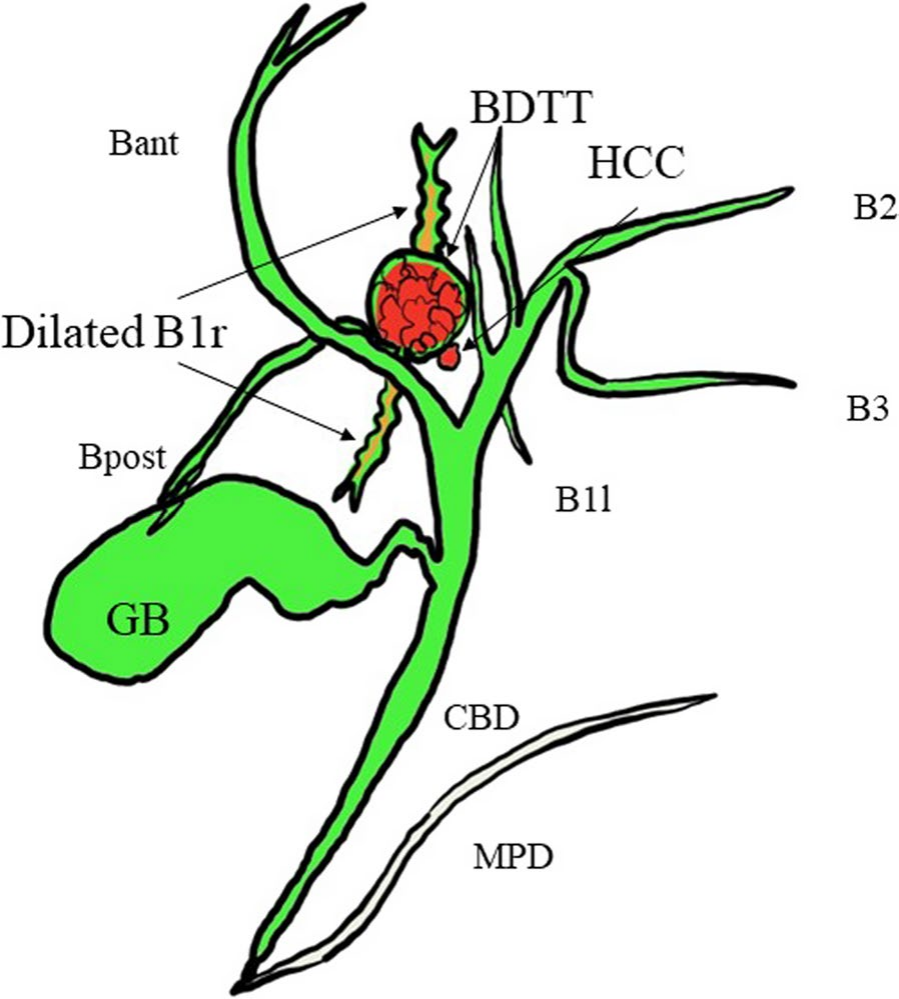
Schema of the relationship between the biliary tree, HCC and BDTT. A 2 mm HCC has invaded the adjacent bile duct of the caudate lobe and formed a 2 cm BDTT. The B1r upstream of BDTT was locally dilated

Macroscopically, the lesion was an 18 × 15 mm tumor occupying a dilated intrahepatic bile duct near the right hepatic duct, with a soft, fine papillary tumor (Fig. 4). Histologically, the tumor grew in a nearly cord-like tubular structure and showed carcinoma-equivalent atypia with fine granular, highly acidophilic, abundant cytoplasm, and irregular round nuclei with agglutinated chromatin, consistent with moderately differentiated HCC (Fig. 5). Immunostaining showed that the tumor cells were hepatocyte-positive, Glypican3-negative, HSP70-positive, and CK19-positive suggesting hepatocellular carcinoma. In addition, a 2 mm-sized hepatocellular carcinoma was observed in the liver parenchyma near the bile duct, where the main tumor was located (Fig. 6). Based on these findings, we have diagnosed the tumor as HCC with BDTT, rather than as cholangiocarcinoma (T2N0M0 Stage II; TNM classification of malignant tumors; eighth edition [6]). The cystic lesion seen on the preoperative images was considered as a dilated bile duct filled by BDTT, and low attenuation component in the lesion was thought to be a gap between the bile duct and the tumor thrombus. Although mild fatty changes were observed in the non-cancerous liver parenchyma, no fibrotic changes were observed. Based on these pathological and preoperative imaging findings, a schema of the relationship between the biliary tree, HCC and BDTT was illustrated in Fig. 7. The patient recovered well with no signs of recurrence 20 months after surgery.

## Discussion

BDTT is a relatively rare but well-known manifestation of HCC. According to the 22nd National Primary Liver Cancer Follow-up Survey Report in Japan (2012–2013), the frequency of bile duct tumor thrombus was 3.4% by imaging diagnosis in all registered cases, including operated and non-operated cases, and 3.2% according to pathological findings [7]. This is lower than the frequencies of portal vein tumor thrombus (imaging, 13.9%; operative findings, 15.9%) and hepatic vein tumor thrombus (imaging, 4.6%; operative findings, 6.5%), indicating that this is a relatively rare form of invasion.

Clinical manifestations of BDTT are often obstructive jaundice and biliary hemorrhage [5, 8]. Jaundice is characterized by the spontaneous resolution of intrahepatic cholangiocarcinoma due to tumor growth and necrosis in the bile duct, and increased intraductal bile duct pressure may be caused by the induction of intraductal pressure due to the induction of tumor necrosis and jaundice, as well as stone-like symptoms, such as pain and vomiting [9].

Despite the remarkable progress in diagnostic imaging, it is often difficult to differentiate hepatocellular carcinoma with intraductal growth of the bile duct, especially in cases with obstructive jaundice as the initial symptom and no obvious intrahepatic mass, from hilar bile duct carcinoma or intraductal growth of the bile duct-type hepatoma [10]. Correct preoperative diagnosis has been reported in only 26.5% of cases, indicating the challenges of clinical diagnosis [5]. In the present case, intrahepatic intraductal papillary neoplasm of the bile duct was considered as the primary diagnosis, because the preoperative imaging showed no obvious cirrhosis, the bile ducts were dilated at the periphery of the mass, and the contrast pattern of the tumor was not typical for HCC. The tumor initially appeared to have an internal cystic component, although it was a space between the BDTT and the bile duct wall, and the AFP and PIVKA-II were within the standard values.

Several hypotheses have been proposed regarding the mechanism underlying BDTT development [5]. A distal tumor may grow continuously until it fills the entire extrahepatic biliary system; a fragment of necrotic tumor may separate from the proximal intraductal growth, migrate to the distal common bile duct, and cause an obstruction, and eventually hemorrhage from the tumor may partially or completely fill the biliary tract with tumor-containing blood clots [4, 11–14]. In the present case, although the tumor was small (2 mm), it is possible that the necrotic tissue of the HCC originating in the hepatic parenchyma near the right hepatic duct may have migrated and developed into BDTT at the right hepatic hilum. As recent studies suggest that HCC with BDTT may arise from liver stem/progenitor cells in the canals of Hering particularly when the primary lesion is very small [15, 16], it is possible that the tumor in this case originated from such a mechanism.

Pathological characteristics include a large tumor diameter, portal vein invasion, often with intrahepatic metastases, and intermediate or poorly differentiated histology [17]. The 5-year survival rate after curative resection is 48%, and poor prognostic factors include tumor size greater than 5 cm, positive vascular invasion, and cirrhosis [8]. As these factors are not compatible with the present case, long-term survival was expected.

## Conclusions

In conclusion, we encountered an extremely rare case of small HCC with gross BDTT that was completely resected by right hemihepatectomy. To the best of our knowledge, no similar reports have been published previously. It is important to include micro-HCC with BDTT as one of the differential diagnoses when localized intrahepatic bile duct dilatation is observed, especially in patients with a history of hepatitis virus infection or fatty liver disease.

## Data Availability

Not applicable.

## Abbreviations

HCC: Hepatocellular carcinoma
BDTT: Bile duct tumor thrombus
AFP: Alpha-fetoprotein
PIVKAII: Protein induced by vitamin K absence or antagonist-II
CECT: Contrast-enhanced computed tomography
